# Sociodemographic and clinical profiles comparison in an acute hospital unit after a decade (2006-2007 vs 2017-2018)

**DOI:** 10.1192/j.eurpsy.2021.991

**Published:** 2021-08-13

**Authors:** M. Peraire, C. Guinot, M. Villar

**Affiliations:** Castelló, Consorcio Hospitalario Provincial de Castellón, Castelló de la Plana, Spain

**Keywords:** Psychiatric diagnosis, epidemiology, acute unit, sociodemographic and clinical changes

## Abstract

**Introduction:**

It has been recently proposed that diagnoses traditionally framed in axis II of the DSM and diseases related to the elderly are progressively replacing serious mental illness in acute inpatient wards.

**Objectives:**

To study the clinical and sociodemographic characteristics of the patients in an acute psychiatric unit, and to compare them between a ten-year period.

**Methods:**

Observational, descriptive, and retrospective study that analyzes the data recorded in the discharge reports from the acute ward of the Hospital Provincial de Castellón.

**Results:**

Among the studied patients, we found statistically significant differences regarding gender, age, readmission rate, and stay duration between the two periods. In the most recent one (2017-18), more women and elderly have entered, with shorter stays and fewer readmissions. In both periods, the most prevalent psychiatric diagnoses are by far serious mental illness (bipolar disorder, schizophrenia). By grouping the diagnoses into five broad categories (serious mental illness, dementias, personality disorders, drug misuse, and others), we found significant differences in their distribution. Lately, more personality disorders and dementias were admitted as the main diagnosis, while serious mental illness and substance use disorders increased their prevalence as accessory diagnoses.

**Conclusions:**

The research carried out allows us to conclude that the clinical and sociodemographic profile of patients admitted to an acute unit is changing. It would be advisable to investigate the causes that motivate it and modify the devices to adapt to this new reality.

